# Home learning environment and out-of-home activities: their relations to prosocial behaviour and peer relationships in primary school children

**DOI:** 10.1007/s12144-022-03410-6

**Published:** 2022-07-25

**Authors:** Anna Volodina

**Affiliations:** University of Bamberg, Markusplatz 3, 96047 Bamberg, Germany

**Keywords:** Prosocial behaviour, Peer relationships, Home learning environment, Out-of-home activities, Primary school

## Abstract

There is consensus that child socio-emotional development is influenced by various contexts, such as the family one. Research on influencing factors on child socio-emotional skills mainly investigated the effects of home learning environment, whereas the effects of out-of-home activities were often analysed mainly in samples of adolescents. The present study aimed to shed light on effects of preschool home learning environment and out-of-home activities on two facets of socio-emotional skills at the beginning of primary school: Prosocial behaviour and peer relationships. The information on the child prosocial behaviour and peer relationships at preschool age was included with the aim to control for most of the differences across children. Using data from a large sample of children (*N* = 1,818; *M*_age_ = 7.08 years, *SD* = 0.15; 49.9% girls), results of regression analyses show significant effects of out-of-home activities on prosocial behaviour after controlling a range of child- and family-related influencing factors on prosocial behaviour as well as prosocial behaviour at preschool age. The effects of home learning environment were significant after controlling a range of child- and family-related influencing factors on both facets of socio-emotional skills but became nonsignificant after taking into account respective behaviour at preschool age. The results of the present study suggest that fostering participation in out-of-home activities might contribute to an increase of prosocial behaviour in primary school children.

Children’s early socio-emotional skills seem to be rather stable (e.g., Frogner et al., 2021; Keiley et al., 2000). However, socio-emotional skills are longer socially malleable in comparison to cognitive skills (e.g., Heckman et al., 2006). Low levels of socio-emotional skills at childhood are related to, amongst others, poor physical (e.g., obesity) and mental health, poor behavioural outcomes in adulthood (e.g., juvenile delinquency, criminality, substance abuse; Attanasio et al., 2020; Goodman et al., 2000; Reiss, 2013), as well as to poor school performance (e.g., Frogner et al., 2021; Sayal et al., 2015).

Previous research provided evidence on relations between various activities at home and socio-emotional skills of young children (e.g., Rose et al., 2018; Wirth et al., 2020, 2022). Apart from attending formal child care, young children are often involved in out-of-home activities. To date, relations between participation in out-of-home activities and socio-emotional skills were often investigated in samples of adolescents with inconsistent results. For instance, in their review, Farb and Matjasko (2012) reported mixed relations between psychological adjustment (as measured by, amongst other constructs, adolescents’ feelings about themselves and relationships with others) in samples of adolescents aged 12–19 years. Studies on effects of out-of-home activities on socio-emotional skills in primary school children are scarce (e.g., Meroni et al., 2021), but important as out-of-home activities represent an opportunity to develop socio-emotional skills (e.g., Masten & Coastworth, 1998) and, possibly, can reduce socioeconomic inequalities in socio-emotional skills, which have been shown to exist already in 3-year-old children (e.g., van Poortvliet, 2021).

The investigation of socio-emotional skills in childhood is important as this period is a sensitive one in which problematic developmental pathways are considered to be most amenable to change (Shonkoff & Phillips, 2000). Amongst others, the establishment of peer relationships and the acquisition of socially appropriate conduct are considered to be the key developmental tasks of middle childhood (e.g., Masten & Coatsworth, 1998). For instance, various out-of-home activities can facilitate developmental need for social relatedness and offer experiences of teamwork, which might be related to the development of these skills (e.g., Eccles et al., 2003; Masten & Coatsworth, 1998). The present study focusses on two facets of socio-emotional skills (i.e., prosocial behaviour and peer relationships). The need to investigate peer relationships stems from findings on relations of poor peer relationships to internalising (e.g., depressive symptoms, low self-esteem) and externalising (e.g., delinquency, substance abuse) problems in adolescence and adulthood (e.g., Rubin et al., 2015), poor performance at school, school dropout (e.g., Hartup & Moore, 1990), and reduced employment opportunities as well as success at work (e.g., Masten et al., 2010). As to early prosocial behaviour, it is also associated with, amongst others, good school performance and low levels of externalising problems; furthermore, it is related to peer acceptance (e.g., Eisenberg et al., 2015; Frogner et al., 2021).

The present study considers the effects of both out-of-home activities and home learning environment (HLE) for prosocial behaviour and peer relationships. The information on past values of child prosocial behaviour and peer relationships was included with the aim to capture both unobservable characteristics of the child and unobservable past inputs (see Meroni et al., 2021, for similar approach).

## Factors influencing early socio-emotional outcomes

### Theoretical approaches

According to bioecological models of human development, child development is driven by proximal and structural factors at the level of the family as well as by more distal factors (e.g., general beliefs, laws) at the level of the society (Bronfenbrenner, 1986; Bronfenbrenner & Morris, 2006). Drawing on assumptions of bioecological models, specific theoretical accounts aiming to better understand factors influencing the development of socio-emotional skills were developed. For instance, the bioecological model (Bronfenbrenner, 1986; Bronfenbrenner & Morris, 2006) has been adapted by Cicchetti and Lynch (1993) to provide a theoretical framework to examine child socio-emotional outcomes within interacting social systems. In this model, the ontogenic system involves characteristics related to children’s cognition, language, and behaviour. The macrosystem encompasses cultural values and beliefs, while the microsystem refers to the physical and social aspects of the child’s family environment. The exosystem includes elements of Bronfenbrenner’s mesosystem and consists of factors external to children’s immediate environment (e.g., parental employment). Chronosystem refers to the effects of time (longitudinal dimension) on child development (Bronfenbrenner, 1986; Bronfenbrenner & Morris, 2006). Furthermore, Vygotsky (1962, 1978) emphasized the role of social and cultural environment for the development of children’s outcomes.

#### Home learning environment, out-of-home activities, and socio-emotional skills

HLE is a multifaceted construct which consists of a range of various shared routines at home, including, for instance, joint reading activities (e.g., Niklas & Schneider, 2017; Rodriguez & Tamis-LeMonda, 2011). With regard to indicators of HLE, especially shared book reading seems to be particularly relevant for (the development of) socio-emotional skills as it provides opportunities to talk with children about characters’ emotional states and social interactions (e.g., Aram & Aviram, 2009; Kohm et al., 2016; Wirth et al., 2020). Amongst others, storybook reading time has been related to increased social interactions between children and their engagement in more prosocial behaviour (Kohm et al., 2016). Regarding other home learning activities, music making, including joint singing, might be related to socio-emotional skills through effectively satisfying inherent human needs to share experiences, activities, and emotions with others by, for instance, keeping a constant audiovisual representation of the collective intention and shared goal of vocalizing (Kirchner & Tomasello, 2010).

Recently, several studies investigated associations between HLE and socio-emotional skills. In a cross-sectional study, Wirth et al. (2020) reported significant associations between families’ shared reading habits and socio-emotional skills of 3-year-old children. In a longitudinal study, Wirth et al. (2022) documented relations between HLE (e.g., frequency of reading, onset of reading) and children’s socio-emotional skills via their language skills. In another longitudinal study, Rose et al. (2018) found effects of HLE (e.g., frequency of shared book reading) at the age of three years on socio-emotional skills of 8-year-old children. Analysing data from the United Kingdom Millennium Cohort Study, Del Bono et al. (2016) found significant effects of maternal educational (e.g., reading to the child, engaging with the child’s teachers and school initiatives) and recreational (e.g., outdoor recreation, indoor games) time inputs on both cognitive and socio-emotional (i.e., operationalized by the Total Difficulty Score of the Strength and Difficulties Questionnaire [SDQ; Goodman, 1997]) skills of children between ages three and seven. With regard to socio-emotional skills, this long-term impact disappeared after skill persistence has been accounted for.

Few studies investigated the effects of both “analog” and “digital” (i.e., shared activities between children and parents while using digital devices as well as the time spent on these activities; Segers & Kleemans, 2020) HLE on socio-emotional skills. For instance, using data from nationally representative German sample, Lehrl et al. (2021) found associations between analog HLE activities with better socio-emotional skills of preschoolers, while associations between digital HLE activities with socio-emotional skills of preschoolers were negative (i.e., high frequency of joint digital media-related activities was associated with low prosocial behaviour and more difficult behaviour as measured by the SDQ). Thus, scarce research on relations between indicators of HLE and socio-emotional skills mainly suggests the existence of significant positive associations between them, especially in case of analog HLE-activities.

Structured activities are usually defined as those which are “organised by adults around specific social or behavioural goals” (Fletscher et al., 2003, p. 642) and include, amongst others, children’s involvement in sport activities and music lessons (Fletscher et al., 2003). Amongst others, out-of-home activities provide opportunities to form strong social bonds with peers (e.g., Eccels et al., 2003). Fletscher et al. (2003) reported associations between frequent participation in sport activities and social competence (as assessed by their teachers using the Harter Perceived Competence; Harter, 1982) of 147 students from Grade 4 in the United States, but no associations of activity participation (i.e., the number of sport teams, the number of church activities, and the number of clubs participated in by each child) with externalising or internalising behaviour as measured by the Child Behaviour Checklist (CBCL; Achenbach & Edelbrock, 1981) completed by their parents and teachers. Furthermore, Metsäpelto and Pulkkinen (2012) found associations between participation in arts, crafts, and music activities, with a higher adaptive behaviour (i.e., socially active behaviour, constructiveness, and compliance) in children aged 9 to 10 years. In a similar vein, Molinuevo et al. (2010) found out-of-home activities (grouped into categories “sports” and “nonsports”) to be related to better emotional and behavioural adjustment (as assessed by parents and teachers using the SDQ) and social competence (as measured by subscales from the School Social Behaviour Scales completed by teachers; Merrell, 2002) in primary school children between 6 and 12 years old in a large Spanish sample. In the study by Molinuevo et al. (2010), sports activities were slightly more relevant for boys, whereas nonsports activities were more related to measures of adjustment in girls. Portela-Pino et al. (2021) found participation in sport activities of adolescents aged between 11 and 18 years to be related to a lower level of socio-emotional skills and suggested the share of negative emotions (e.g., competitiveness, frustrations) which often occur within sport activities as a possible explanation of this result. Thus, overall, participation in out-of-home activities seems to have positive effects for socio-emotional skills in primary school children. However, studies which investigated effects of out-of-home activities on socio-emotional skills rarely considered indicators of HLE.

Using time use diaries from the Longitudinal Study of Australian Children, Fiorini and Keane (2014) analysed time use of about 1,000 children aged between four and nine years and described the allocation of their time into a range of activities (e.g., educational activities, social activities, general care). They found educational activities (e.g., time spent helping with chores, talked to, reading a story) to be the most relevant for cognitive skills, while socio-emotional skills (e.g., index of behavioural problems, index of good relationships with others, index of emotional problems) were uncorrelated to diverse types of time allocations. Investigating influencing factors on socio-emotional adjustment of 8-9-year-old children from the Longitudinal Study of Australian Children, Sanson et al. (2011) found children’s engagement in out-of-home activities (e.g., visiting libraries, attending sport events with a family member) at 4–5 and 6–7 years as well as involvement in within-home-activities (e.g., reading to the child, arts and crafts activities) at 6–7 years to be related to better socio-emotional adjustment as measured by the composite score from all five SDQ-subscales. In line with findings by Sanson et al. (2011), analysing data from the United Kingdom Millennium Cohort Study, Meroni et al. (2021) found positive effects of participation in various out-of-home activities (e.g., sports) and active time with parents on prosocial behaviour and peer relationships of 11-year-old children as measured by the SDQ. Thus, previous research has rarely simultaneously considered both HLE and out-of-home activities when investigating influencing factors on socio-emotional skills and existing evidence on effects of out-of-home activities (in case these are considered simultaneously with HLE) on socio-emotional skills is inconclusive.

#### Other influencing factors on socio-emotional skills

Apart from HLE and out-of-home activities, a range of factors is associated with socio-emotional skills both theoretically (e.g., in bioecological models of development) and empirically. With regard to results of empirical studies, children from families with high socioeconomic status are usually considered to be more socially competent and have low levels of externalising as well as internalising problems than children from families with low socioeconomic status (e.g., Fletscher et al., 2003). For instance, it has been consistently shown that children from high-income families and/or whose parents are high-educated demonstrate comparatively better socio-emotional skills as early as three years of age than children from low-income families and/or with low-educated parents (e.g., Brooks-Gunn & Duncan, 1997; Bradshaw & Holmes, 2008; Davis et al., 2010; Kiernan & Huerta, 2008; Newton et al., 2014; Reiss, 2013; Reiss et al., 2019). Furthermore, children growing up with both parents tend to have better socio-emotional skills than those who grow up with a single parent (e.g., Downey et al., 2015; Kalff et al., 2001). Children with migration background often have externalising behaviour problems (e.g., Kalff et al., 2001). However, other studies reported comparable levels of socio-emotional skills for children with and without migration background (e.g., Han, 2010; McNally et al., 2019). Furthermore, as argumented by Brody (1998), having siblings provides vast opportunities for social learning and development through extensive interactions. In line with this argument, having siblings has been associated with better social skills (e.g., Brody, 1998, 2004; Downey & Condron, 2004; Downey et al., 2015).

Formal child care attendance and maternal employment are not completely independent from each other (e.g., Côté et al., 2013), as, for instance, children of working mothers often attend formal child care. In Germany, enrolment rates of zero-to-two-year-old children in formal child care in 2018 were 33.6% (German Federal Statistical Office, 2018), while 74.7% mothers with a least one child aged under 18 years were in employment in 2019 (German Federal Statistical Office, 2022). Formal child care attendance is usually related to better children’s cognitive skills (e.g., Berger et al., 2021; NICHD Early Child Care Research Network, 2002; Sammons et al., 2004; van Huizen & Plantega, 2018). For instance, with regard to language skills, it is assumed that high quality caregiver-child verbal interactions in formal child care along with learning stimulation may serve as a buffer from poor language outcomes for children with lower quality language experiences at home (e.g., Dearing et al., 2009; Votruba-Drzal et al., 2013). Comparing to studies on effects of formal child care attendance on cognitive skills, results with respect to the effects of very early formal child care attendance on socio-emotional skills in early childhood when children’s socio-emotional skills are still rather restricted are not clear-cut (e.g., Barnett, 1995; Berger et al., 2021; Linberg et al., 2019; van Huizen & Plantega, 2018). For instance, many studies documented an increased risk for having low socio-emotional skills for children who start to attend formal child care from very early on (e.g., at the age of one or two years) and/or spend many hours (often more than 30 h) in formal child care from early on (e.g., Belsky, 2002; Berger et al., 2021; Bradley & Vandell, 2007; NICHD Early Child Care Research Network, 2002). However, small positive effects of the formal child care attendance of any type at nine months on socio-emotional skills at the age of 5 years were also documented (Russell et al., 2016). Furthermore, the fact of attending early formal child care more than one year under the age of three years was associated with lower rates of peer problems as rated by parents – but not with prosocial behaviour – of 3-year-old children in Germany (Linberg et al., 2019), while later entry in formal child care (i.e., later than the age of 29 months) was related to increase of parental-rated peer problems and reduction of prosocial behaviour of 7-year-old children in the United Kingdom (Peter et al., 2016). Notably, there are studies which found no effects of formal child care attendance on socio-emotional skills (e.g., Del Bono et al., 2016, investigating socio-emotional skills of 7-year-old children in the United Kingdom). Negative effects of early attendance of formal child care might be related to stress associated with large group size with few supervision by adults (e.g., Votruba-Drzal et al., 2013), while positive ones may be associated with a range of interaction opportunities with peers (e.g., playing together). As in case of studies investigating relations between attendance of formal child care and children’s socio-emotional skills, results of studies investigating associations between maternal employment and child socio-emotional skills are inconclusive. For instance, Sanson et al. (2011) reported negative effect of having a mother working full time (relative to not being in the labour force) for socio-emotional skills of 8-9-year-old children. However, Schoon et al. (2021) found no significant associations between parental worklessness (i.e., neither parent in employment) and peer relationships of 5-year-old children.

Regarding child characteristics, child reactive temperament (e.g., volatile, quick to anger) is often related to low social competence (see Sanson et al., 2004, for an overview). Furthermore, children with greater nonverbal intelligence show, amongst others, better cooperation skills (Rose et al., 2018). Vygotsky (1962) suggested strong relations between language and thought, arguing that self-directed speech is related to, amongst others, social-cognitive problem solving. Relations between child language skills and socio-emotional skills have been shown cross-sectionally and longitudinally (e.g., Rose et al., 2018; Wirth et al., 2020, 2022). Furthermore, gender differences in child socio-emotional skills have often been documented (e.g., Linberg et al., 2019; Newton et al., 2014; Sanson et al., 2011; Wirth et al., 2020, 2022), with girls usually being rated more prosocial and having better peer relationships than boys. In addition, boys and girls tend to be involved in distinct types of out-of-home activities, with boys often preferring sport activities and girls, arts and music activities (e.g., Eccles et al., 2003; Metsäpelto & Pulkkinen, 2012). Moreover, girls tend to participate in more total activities and in a wider range of activities compared to boys (Eccles et al., 2003).

#### Research questions

The aim of the present study is to explore if and how out-of-home activities and HLE contribute to two facets of socio-emotional skills – prosocial behaviour and peer relationships – in primary school children. In particular, the present study examines following research questions: (1) Are HLE and out-of-home activities related to prosocial behaviour and peer relationships in primary school children when considered simultaneously? (2) Do effects persist if information on prosocial behaviour and peer relationships at preschool age is taken into account?

When investigating relations between HLE, out-of-home activities, prosocial behaviour, and peer relationships, various child and family-related characteristics need to be considered. In the present study, family structure, the presence of siblings, SES of the family, number of books at home, migration background, maternal work and attendance of formal child care when the child was two years old, nonverbal abilities, child language skills as well as child gender and age are considered. Furthermore, prior levels of prosocial behaviour and peer relationships are controlled for with the aim to disentangle developmental benefits gained from the participation in out-of-home activities from pre-existing differences.

## Method

### Participants

The present study used data of Starting Cohort 1 of the German National Educational Panel Study (NEPS-SC1; Blossfeld et al., 2011). In the NEPS-SC1, a representatively drawn national sample of about 3,500 children born from February to June 2012 is followed longitudinally from 7 months of age onwards (Weinert et al., 2016; Zinn et al., 2018). The present study considered 1,818 children with scores on socio-emotional skills in wave 8 (*M*_age_ = 7.08 years, *SD* = 0.15; 49.9% girls).

The NEPS is conducted under the supervision of the German Federal Commissioner for Data Protection and Freedom of Information (der Bundesbeauftragte für den Datenschutz und die Informationsfreiheit; BfDI) and in coordination with the German Standing Conference of the Ministers of Education and Cultural Affairs (Kulturministerkonferenz; KMK), and – in the case of surveys at schools – the Educational Ministries of the respective federal states. The data protection unit of the Leibniz Institute for Educational Trajectories (LIfBi) carefully checks all data collection procedures and materials. Participation in the NEPS study is voluntary and based on the informed consent of participants, which can be revoked at any time. All parents of the NEPS-SC1 give their agreement for participation and answering questions during the assessments as well as a written consent for their children to, e.g., participate in direct assessments.

### Measures

#### Children’s socio-emotional skills

At the average age of 5.11 (*SD* = 0.15) and 7.08 (*SD* = 0.15) years, children’s socio-emotional skills were measured using an age-appropriate parent version of the SDQ, an internationally recognized standard measure used in large-scale surveys (Goodman, 1997). In particular, in the present study, two subscales (i.e., prosocial behaviour [e.g., “Shares readily with other children (treats, toys, pencils, etc.).”] and peer problems [e.g., “Rather solitary, tends to play alone.”]) were used. For the aims of the present study, the items assessing peer relationship problems have been reversed, and the subscale has been renamed into peer relationships. The five items per subscale were answered by the parent. Items are scored on a 3-point Likert scale (0 = *not true*, 1 = *somewhat true*, 2 = *certainly true*). Reliabilities (Cronbachs’ Alpha) were 0.57/0.62 for scale peer relationships and 0.60/0.65 for prosocial behaviour at the average age of 5 and 7 years, respectively.

#### Children’s temperament

As an indicator of a difficult child temperament, the scale Negative Affectivity of the Infant Behaviour Questionnaire Revised (IBQ-R; Gartstein & Rothbart, 2003; Vonderlin et al., 2012) was used. For this scale, parents report the child’s tendency to show negative affect in reaction to displeasing situations. For example, they answered questions such as ‘If you are busy doing something else and child is unable to get your attention, how often does he/she end up crying?’ on a 7-point Likert scale (0 = *never*, 6 = *always*). The three items indicating negative affectivity at the child average age of 0.59 (*SD* = 0.06) months, of 1.13 (*SD* = 0.12), and of 2.22 (*SD* = 0.10) years were used to generate a composite score for difficult temperament (Cronbachs’Alpha = 0.53, 0.53, and 0.54, respectively), indicating the mean tendency to show comparatively more and stronger negative affect during the first three years of life.

#### Children’s language skills

Receptive vocabulary was measured at the average age of 5.11 (*SD* = 0.15) years using a German version of the Peabody Picture Vocabulary Test (PPVT-IV; Lenhard et al., 2015) presented by a tablet-computer. The test includes 228 items (i.e., 19 sets with 12 items each with a stopping rule according to the number of wrong/no answers within a set) in ascending difficulty. For each item, the child is presented with four pictures and has the task to identify which of these matched the orally presented word. The total number of correct items was used in the analyses.

#### Children’s nonverbal abilities

As an indicator of children’s nonverbal reasoning skills, the subtest Categories from the Snijders-Oomen Nonverbal Intelligence Test for 2½-to7-Year-Old Children (SON-R 2 1/2–7; Tellegen et al., 2005) was administered when children were on average 3.21 (*SD* = 0.09) years old. Children had to categorise different generic concepts by sorting various pictures (Cronbachs’Alpha = 0.84). In analyses, scaled scores (i.e., weighted likelihood estimate) were used.

#### Socioeconomic status

In the present study, parental education, occupation, and family income at the child average age of 7.08 (*SD* = 0.15) years were used to build an indicator of family socioeconomic status. With regard to parental education, the present study includes a measure of parental education based on the highest level of education attained by a parent who is living with the child. The levels of education were categorised using the International Standard Classification on Education (ISCED). Parents also provided information on their occupation. In the present study, the highest International Socio-Economic Index of Occupational Status in the family was used to measure parental occupational status (Ganzeboom & Treiman, 1996). Regarding income, parents provided their net income. For the analyses, the highest parental education, occupation, and family income were first z-standardized and then combined into an indicator of socioeconomic status (see Wirth et al., 2022, for a similar approach).

#### Family structure

In the present study, distinction was made between children who live in a one- or two-parent household (0 = *one-parent household*, 1 = *two-parent household*) at the child average age of 7.08 (*SD* = 0.15) years.

#### Number of siblings

In the present study, the number of siblings living with the child regardless of their relationship (i.e., it includes full, half, step and foster siblings, etc.) at the child average age of 7.08 (*SD* = 0.15) years was considered.

#### Migration background

As an indicator of migration background, in the present study, a binary indicator for whether any language other than the majority language is spoken in the child’s home (0 = *other language(s) than the majority language is spoken at home/majority and other language(s) than the majority language are spoken at home*, 1 = *only majority language is spoken at home*) at the child average age of 7.08 (*SD* = 0.15) years was used.

#### Home learning environment

In the present study, the frequency of joint book reading, music making, sport activities, handicrafts, puzzle making, and role games at the average age of 5.11 (*SD* = 0.15) years was considered. Parents were asked how often they or someone else in their home jointly engage in e.g., picture book reading with the child (1 = *never* to 8 = *several times a day*). In analyses, a mean score of joint (analog) activities at home was used as an indicator of HLE (CFI = 0.951, RMSEA = 0.066; Cronbachs’Alpha = 0.68).

#### Number of books

Parents provided information on the number of books at home 1 = *0–10*, 6 = *more than 500 books*) when their children were on average 26 months old.

#### Digital home learning environment

When children were on average 6.17 (*SD* = 0.13) years old, parents provided information on the frequency of digital media use by children themselves on a 5-point-Likert scale ranging from 1 = *rarely than once in 2–3 weeks or never* to 5 = *everyday*.

#### Out-of-home activities

When children were on average 6.17 (*SD* = 0.13) years old, parents reported on their attendance of four activities (i.e., sport, dance lessons, music lessons, and foreign language lessons; 1 = *yes*, 0 = *no*). In analyses, a sum score of the use of out-of-home activities was considered.

#### Attendance of formal child care

In the present study, an indicator for exposure to formal child care (i.e., nursery, daycare centre) in the second year of a child’s life (i.e., 0 = *no attendance of formal child care*, 1 = *attendance of formal child care*) was created.

#### Maternal employment

As indicator of maternal employment, the information on whether the child’s mother had worked during the second year of the child’s life (0 = *no*, 1 = *yes*) was used.

#### Child gender and age

In the present study, information on children’s gender (0 = *girl*, 1 = *boy*) and child age in days was considered.

### Statistical procedures

#### Regression analyses

Regression analyses were used with the aim to investigate relations between HLE, out-of-home activities, and socio-emotional skills (i.e., prosocial behaviour and peer relationships). In particular, in the first model, effects of HLE, out-of-home activities as well as of covariates on prosocial behaviour and peer relationships were considered. In the next step, respective socio-emotional skills at the child average age of 5.11 (*SD* = 0.15) years were taken into account.

#### Missing data

Multiple imputation as implemented in STATA 16 (Raghunathan et al., 2001; 50 datasets) was used to account for missing information in the independent variables. All variables included in the analyses were used for the imputation. The results of the subsequent analyses with 50 imputed datasets were automatically combined in STATA 16 in accordance with formulas proposed by Rubin (1987).

## Results

### Descriptive statistics

Descriptive statistics of study variables are shown in Table 1, whereas results of correlational analyses are presented in Table 2. There were significant associations of prosocial behaviour at the mean age of 7 years with child gender, family structure, negative affectivity, nonverbal abilities, HLE, and out-of-home activities, suggesting that girls, children in families with two parents, low negative affectivity, high nonverbal abilities, and those who profit from (frequent) HLE and out-of-home activities have high levels of prosocial behaviour. With regard to peer relationships at the mean age of 7 years, significant correlations emerged between peer relationships and child gender, socioeconomic status, migration background, family structure, number of siblings, maternal work, language skills, nonverbal abilities, HLE, number of books at home, and out-of-home activities. Thus, children with good peer relationships are often girls, live with two parents and have siblings, have families with high socioeconomic status and do not have migration background, have mothers who were at work when children were 2 years old, have high receptive vocabulary, nonverbal abilities, large amount of books at home, and profit from (frequent) HLE- and out-of-home activities. Furthermore, prosocial behaviour and peer relationships at the mean age of 7 years showed high correlations with respective behaviours at the mean age of 5 years. Finally, small correlations emerged between prosocial behaviour and peer relationships when the child was on average 5.11 (*SD* = 0.15) and 7.08 (*SD* = 0.15) years old.

**Table 1:** Descriptive statistics for study variables

Variables	Categories					
Gender		*M*	*SD*	Min	Max	%
	male					50.06
	female					49.94
Age (in days)		2585.81	51.98	2453	2710	
Socioeconomic status		0.10	0.79	−2.44	3.76	
Migration background						
	only German					80.15
	not only German					19.85
Family structure						
	two-parent household					81.62
	one-parent household					18.38
Number of siblings		1.16	0.83	0	9	
Attendance of center-based care						
	yes					74.12
	no					25.88
Maternal work						
	yes					65.73
	no					34.27
Language skills		90.58	20.41	30	181	
Negative affectivity		3.86	0.91	0.5	6	
Nonverbal abilities		0.27	2.50	−4.06	6.09	
Home learning environment		6.40	0.80	2.67	8	
Number of books		4.10	1.00	1	6	
Use of digital media		3.47	1.27	1	5	
Out-of-home activities		1.26	0.84	0	4	
Prosocial behaviour (5 years)		7.93	1.55	1	10	
Peer relationships (5 years)		6.94	1.34	0	8	
Prosocial behaviour (7 years)		7.94	1.65	1	10	
Peer relationships (7 years)		6.79	1.49	0	8	

**Table 2:** Results of correlational analyses

Variables	(1)	(2)	(3)	(4)	(5)	(6)	(7)	(8)	(9)	(10)	(11)	(12)	(13)	(14)	(15)	(16)	(17)	(18)
(1) Gender^a^	1																	
(2) Age	0.01	1																
(3) SES^b^	−0.02	0.00	1															
(4) Migration background^c^	0.02	−0.02	0.20*	1														
(5) Family structure^d^	−0.03	−0.04	0.24*	−0.03	1													
(6) Number of siblings	−0.01	−0.01	0.02	−0.08*	0.16*	1												
(7) Formal child care^e^	−0.03	0.02	0.09*	0.01	−0.04	0.02	1											
(8) Maternal work^f^	0.02	−0.03	0.23*	0.11*	−0.00	−0.20*	0.08*	1										
(9) Language skills	0.17*	−0.10*	0.34*	0.25*	0.06*	−0.11*	0.01	0.10*	1									
(10) Negative affectivity	−0.00	−0.03	−0.03	−0.02	0.00	−0.01	0.03	0.00	−0.05	1								
(11) Nonverbal abilities	−0.14*	−0.03	0.18*	0.05*	0.06*	−0.06*	0.06*	0.09*	0.17*	−0.03	1							
(12) HLE	−0.26*	0.02	0.19*	0.07*	0.10*	0.06*	−0.04	−0.02	0.07*	−0.06*	0.10*	1						
(13) Number of books	−0.04	0.01	0.53*	0.19*	0.13*	0.10*	0.05*	0.12*	0.35*	−0.10*	0.18*	0.28*	1					
(14) Use of digital media	0.04	0.04	−0.05*	−0.04	−0.02	−0.06*	−0.00	0.05*	−0.03	0.03	−0.03	−0.07*	−0.10*	1				
(15) Out-of-home activities	−0.19*	−0.01	0.27*	0.05*	0.11*	−0.07*	−0.01	0.07*	0.06*	−0.05*	0.09*	0.11*	0.20*	−0.08*	1			
(16) Prosocial behaviour (5 years)	−0.18*	0.07*	−0.02	−0.01	0.03	0.03	0.03	−0.02	−0.09*	−0.13*	0.07*	0.21*	0.01	−0.02	0.06*	1		
(17) Peer relationships (5 years)	−0.05*	0.02	0.17*	0.09*	0.02	0.05*	0.05	0.09*	0.07*	−0.06*	0.06*	0.17*	0.11*	−0.03	0.06*	0.11*	1	
(18) Prosocial behaviour (7 years)	−0.19*	0.01	0.02	−0.04	0.09*	0.05	−0.02	−0.01	−0.04	−0.10*	0.07*	0.16*	0.03	0.01	0.11*	0.55*	0.13*	1
(19) Peer relationships (7 years)	−0.09*	0.04	0.23*	0.10*	0.09*	0.06*	0.04	0.10*	0.09*	−0.03	0.10*	0.17*	0.15*	−0.01	0.11*	0.13*	0.48*	0.19*

*N* = 1,818

SES = socioeconomic status; HLE =home learning environment. ^a^Girl = 0, boy = 1. ^b^Socioeconomic status is a mean value from z-standardized indicators of family income, highest parental education, and highest parental occupation. ^c^Not only German = 0, only German = 1. ^d^One-parent household = 0, two-parent household = 1. ^e^No attendance of formal child care at the age of 2 years = 0, attendance of formal child care at the age of 2 years = 1. ^f^Mother not at work at the child age of 2 years = 0, mother at work at the child age of 2 years = 1

*p < .05

### Results of regression analyses

Results of regression analyses are shown in Table 3. With regard to prosocial behaviour, in Model 1, both HLE and out-of-home activities were associated with prosocial behaviour. In addition, the presence of two parents at home and low negative affectivity were related to high levels of prosocial behaviour. Furthermore, girls showed more prosocial behaviour than boys. After the addition of prosocial behaviour at the average age of 5 years (Model 2), negative affectivity and HLE were not more associated with prosocial behaviour at the average age of 7 years, whereas effects of out-of-home activities, child gender, and family structure on child prosocial behaviour remained significant. Prosocial behaviour at the average age of 5 years was the strongest predictor of prosocial behaviour at the average age of 7 years, suggesting considerable stability in individual differences in prosocial behaviour over time.

**Table 3:** Results of regression analyses

	Prosocial behaviour				Peer relationships			
	Model 1		Model 2		Model 1		Model 2	
	β	SE	β	SE	β	SE	β	SE
Child gender^a^	−0.14*	0.08	−0.08*	0.07	−0.04^+^	0.07	−0.04*	0.07
Child age	0.01	0.00	−0.02	0.00	0.04^+^	0.00	0.03	0.00
Socioeconomic status^b^	−0.04	0.06	−0.01	0.05	0.14*	0.06	0.10*	0.05
Migration background^c^	−0.04^+^	0.10	−0.04^+^	0.09	0.07*	0.10	0.04^+^	0.09
Family structure^d^	0.07*	0.11	0.07*	0.09	0.03	0.10	0.04^+^	0.09
Number of siblings	0.04	0.04	0.02	0.04	0.07*	0.04	0.04^+^	0.04
Formal child care^e^	−0.02	0.10	−0.03	0.09	0.02	0.09	−0.00	0.09
Maternal work^f^	0.01	0.09	0.01	0.08	0.07*	0.08	0.03	0.07
Language skills	−0.01	0.00	0.02	0.00	0.02	0.00	0.02	0.00
Negative affectivity	−0.10*	0.04	−0.02	0.04	−0.02	0.04	−0.00	0.04
Nonverbal abilities	0.03	0.02	0.01	0.01	0.04	0.02	0.02	0.01
Home learning environment	0.11*	0.06	0.01	0.05	0.10*	0.05	0.02	0.05
Number of books	−0.02	0.05	−0.01	0.04	−0.00	0.04	0.01	0.04
Use of digital media	−0.00	0.03	−0.00	0.02	−0.02	0.02	−0.02	0.02
Out-of-home activities	0.07*	0.05	0.04*	0.04	0.05^+^	0.04	0.04^+^	0.04
Respective scale at the average age of 5 years			0.53*	0.02			0.45*	0.02
*R* ^2^	0.07		0.32		0.09		0.27	
*∆R*^*2*^			0.25*				0.18*	

*N* = 1,818

SES = socioeconomic status; HLE = home learning environment. ^a^Girl = 0, boy = 1. ^b^Socioeconomic status is a mean value from z-standardized indicators of family income, highest parental education, and highest parental occupation. ^c^Not only German = 0, only German = 1. ^d^One-parent household = 0, two-parent household = 1. ^e^No attendance of formal child care at the age of 2 years = 0, attendance of formal child care at the age of 2 years = 1. ^f^Mother not at work at the child age of 2 years = 0, mother at work at the child age of 2 years = 1

^+^*p* < .10, *p < .05

Regarding peer relationships, in Model 1, HLE was significantly associated with peer relationships at the 5%-level, while association between the out-of-home activities with peer relationships was significant only at the 10%-level. Furthermore, high socioeconomic status, having no migration background and having siblings, as well as having working mother at the age of two years were related to high level of peer relationships. After the addition of peer relationships at the average age of 5 years (Model 2), the effect of out-of-home-activities on peer relationships was still significant at 10%-level, while the effect of HLE on peer relationships became nonsignificant. Furthermore, girls showed better peer relationships than boys and children from families with high socioeconomic status had better peer relationships compared to children from families with low socioeconomic status. Peer relationships at the average age of 5 years were the strongest predictor of peer relationships at the average age of 7 years, suggesting considerable stability in individual differences in peer relationships over time.

## Discussion

In the present study, relations between HLE, out-of-home activities, children’s prosocial behaviour and peer relationships have been investigated. Results of regression analyses revealed significant effects of out-of-home activities for prosocial behaviour after controlling for a range of factors related to prosocial behaviour and for prosocial behaviour at preschool. The effect of out-of-home activities for peer relationships was only significant at 10%-level. Notably, the effects of HLE were only significant in case respective socio-emotional skills at preschool age were not taken into account. These results are partially in line with those by Sanson et al. (2011) and Meroni et al. (2021), who found significant effects of out-of-home activities on socio-emotional skills when investigating effects of HLE and out-of-home activities on socio-emotional skills. However, in contrast to results of studies by Sanson et al. (2011) and Meroni et al. (2021), in the present study, effects of HLE on prosocial behaviour and peer relationships were only significant before respective skills at preschool age have been added. This discrepancy might be explained by, for instance, measurement of socio-emotional skills (e.g., Sanson et al., 2011, used the composite score from all five SDQ-subscales in their analyses) and/or age of children (e.g., Meroni et al., 2021, considered 11-year-old children). Thus, from the policy perspective, the results of the present study call for the provision of out-of-home activities for children in late preschool age with the aim to increase their prosocial behaviour.

The effects of digital activities on socio-emotional skills were not significant. These findings align with those by Meroni et al. (2021), who – although considering socio-emotional skills of 11-year-old children – found a negative effect of video-screen time on emotional problems of 11-year-old children, but no effects on their prosocial skills or peer relationships. Interestingly, Lehrl et al. (2021) found no associations between digital HLE-activities with socio-emotional skills for toddlers, but, for preschoolers, digital HLE-activities were related to weaker socio-emotional skills. These findings might be associated with the fact that, amongst others, interactions with digital media by older children are less often guided by parents (Lehrl et al., 2021), who also vary in the provision of these regulations, and suggest the need to explicitly consider parental regulation of the use of digital media. Notably, Lehrl et al. (2021) distinguished between prosocial behaviour and total difficulties score of the SDQ, while in the present study prosocial behaviour and peer relationships were considered. Furthermore, it might be useful to consider the quality of digital media. For instance, Segers and Kleemans (2020) only found relations between analog (but not digital activities at home such as reading to the child or playing language and word games) with children’s language skills. The authors argued that one reason of the absence of relations between digital HLE and child language skills might lay in the quality of digital HLE (Segers & Kleemans, 2020; see Dore et al., 2020, for similar findings on relations between media use and language skills and interpretation of results). Notably, in their study, there was a relatively high number of missing answers on questions on digital activities at home.

The effects of gender, family structure, and socioeconomic status on child skills in the present study were largely in line with results of previous studies (e.g., Downey et al., 2015; Linberg et al., 2019; Wirth et al., 2020). Furthermore, attendance of formal child care had no significant effects on children’s prosocial behaviour and peer relationships. Investigating effects of attendance of formal child care on socio-emotional skills at age 3;2 in Germany, Linberg et al. (2019) reported – in line with the results of the present study – no significant effects of the duration of the formal child care on prosocial behaviour. However, Linberg et al. (2019) documented those children who attended formal child care longer show less peer problems compared to children who did not attend formal child care at all. Discrepancies with regard to effects of the attendance of formal child care on socio-emotional skills might be due to the consideration of prosocial behaviour and peer relationships in primary school children in the present study and in 3-year-old children in the study by Linberg et al. (2019). With regard to effects of maternal work, in the present study, maternal work at the child age of 2 years did not affect child prosocial behaviour but had an effect on peer relationships in primary school before peer relationships at preschool age were taken into account. The finding of the effect of out-of-home activities on prosocial behaviour in the present study might suggest that involvement in such kind of activities might contribute to the increase of prosocial behaviour in children of working parents. However, implications of the findings of the present study for children of working parents are not straightforward and require further research. In particular, the nature of work is a complex construct, which includes not only the fact of working, but also working time. In fact, the number of working hours rather than employment *per se* seems to be more related to child development outcomes (e.g., Brooks-Gunn et al., 2002; Hill et al., 2005). In its turn, working time is not only the number of hours worked but also (un)predictability of work schedules, regularity of work (Rönka et al., 2017). While few studies documented negative relations between parental nonstandard working time and child socio-emotional skills (e.g., Daniel et al., 2009; Strazdins et al., 2004), positive effects have also been reported (e.g., Barnett & Gareis, 2007, for effects of shifting work on socio-emotional skills of children aged 8 to 14 years). Notably, using sample of parents of children aged 3 to 12 years, Rönka et al. (2017) found parental nonstandard working time (compared to parents in regular daytime work) and work overtime on a short notice to be associated with higher peer problems in the United Kingdom, Finland, and in the Netherlands. Furthermore, in their study, parental nonstandard working time (compared to regular day work) was associated with lower prosocial behaviour in the United Kingdom, but not in Finland or in the Netherlands. The results of the study by Rönka et al. (2017) suggest that relations between parental work and child behaviour might differ, amongst others, as a function of policies and services (e.g., whether parents typically work full or part time, parental opportunities to reconcile family and work, availability of education and care services) aimed to support working parents, as all three countries considered in their study represent a different social regime in Europe. Furthermore, differences might exist with regard to the organisation of children time. However, the aforementioned studies have not considered child participation in out-of-home activities. Thus, with the aim to gain a more nuanced picture on relations between maternal work and child development, future studies might consider various features of parental work and out-of-home activities in examining factors affecting child socio-emotional skills. Furthermore, temporal order of relations between parental work, out-of-home activities, and child socio-emotional skills is unclear and longitudinal studies are required to answer this question (Strazdins et al., 2004).

The current study has several limitations. For instance, only two facets of socio-emotional skills were considered, because, at the child age of seven years, the NEPS-SC1 only includes two scales from the SDQ. Thus, future studies might investigate associations of HLE and out-of-home activities with further facets of socio-emotional skills (e.g., hyperactivity). Furthermore, future studies might benefit from using more sophisticated instruments to measure socio-emotional skills (e.g., amongst others, scales with more items and response options).

In the present study, parental ratings of child socio-emotional skills were used. However, ratings of parents and teachers often differ (e.g., Achenbach et al., 1987; NICHD Early Child Care Research Network, 2002). When rating child behaviour, parents may emphasise more at-home interactions than interactions with other children, whereas kindergarten teachers only focus child interactions with other children in classroom settings (e.g., Downey & Condron 2004; Hinshaw et al., 1992) and school teachers can judge students’ behaviour against of former cohorts of students they have already taught (e.g., Brandt et al., 2021). However, ratings of behaviours are not perfectly objective as, for instance, they depend on individual experience (Brandt et al., 2021). Thus, future studies on child socio-emotional skills could profit from the inclusion of the assessment of socio-emotional skills from parental and teacher’s perspective.

In the present study, only the issue of participation in out-of-home activities has been taken into account. Unfortunately, the data on duration of specific activities was not available but might be a relevant factor when interpreting effects of out-of-home activities on socio-emotional skills (see, e.g., Durlak et al., 2010; Lester et al., 2020, for the quality of afterschool programs). Furthermore, the information on how long children participated in out-of-home activities was also not available. For instance, Metsäpelto and Pulkkinen (2012) found participation in sport activities to be unrelated to socio-emotional skills. However, they reported associations between participation in sport activities and an increase in adaptive behaviour as well as an increase in internalising problems for children whose participation in such activities lasted two or three years. Out-of-home activities differ in their quality and future studies are warranted to consider both quantitative and qualitative aspects of out-of-home activities (see also Durlak et al., 2010; Portela-Pino et al., 2021, for similar recommendations). Furthermore, future studies on relations between HLE, out-of-home activities, and socio-emotional skills might profit from the use of experience sampling method. This method would help to better understand how and with whom children spend their time by collecting data at random intervals through the day (Arndt et al., 2021; Larson & Csikszentmihalyi, 2014).

The data on which the present study relied have been collected before the COVID-19 pandemic. Around the world, the COVID-19 restrictions were implemented in different ways (e.g., few restrictions in Sweden vs. strict restrictions in Spain). In most countries, during lockdowns, educational services were closed, children stayed at home with their parents, while parents had to provide care for their children, home-school them, and perform their own work from home (Benner & Mistry, 2020). Despite differences in the extent of the COVID-19 restrictions, in many countries, children were not able to participate in such out-of-home activities as music lessons, implying that, during the COVID-19 restrictions, HLE might be strongly associated with child behaviour compared to the pre-pandemic time. Results of the study by Egan et al. (2021) suggest that the experience of the negative impact of lockdown in 2020 was not uniform. In particular, Egan et al. (2021) documented that while some parents of children aged 1–10 years in Ireland described negative effects of the closure of ECEC and school settings on children’s socio-emotional development (e.g., lack of interaction with friends, missing out on activities in formal child care and at school), concerns about their own emotional state and children’ socio-emotional behaviour, difficulties in balancing work from home and provide care for their children during the lockdown, others reported positive aspects of lockdown for their children (e.g., more time to play with siblings, lack of routine). Remarkably, Egan et al. (2021) also documented use of screens as ‘digital babysitters’ (p. 930) during the COVID-19 in 2020. In contrast to results by Egan et al. (2021), Liu et al. (2021) reported predominantly negative effects of the COVID-19 pandemic (e.g., high number of behavioural problems in primary school children). Furthermore, Spinelli et al. (2020) found only negative effect of the perception of quarantine on behavioural and emotional problems (i.e., emotional symptoms, conduct problems, and hyperactivity-inattention) of children aged 2–14 years, which was mediated by parental individual (e.g., feeling nervous) and dyadic (e.g., finding it difficult to enjoy interactions with the child) stress. Notably, effects of child experiences with “analog” and “digital” HLE on children socio-emotional skills during the COVID-19 restrictions were not investigated in these studies (i.e., in studies by Egan et al., 2021; Liu et al., 2021; Spinelli et al., 2020). For instance, while parents might have difficulties in reconciling work and care for their children (which might be associated with low frequency of such activities at home as reading together), children might have played prosocial games with their friends. Specifically, researchers recently noted that modern games are complex and dynamic, often including many elements of social interactions, which, in their turn, might be related to prosocial behaviour (e.g., Navarro 2021; Shoshani et al., 2022). With regard to out-of-home activities during the COVID-19 pandemic, physical exercises outside of home might have been favorable for socio-emotional development. In fact, Liu et al. (2021) found regular physical activities during the COVID-19 pandemic to be a protective factor to reduce problematic behaviours during the COVID-19 pandemic. Longitudinal investigation of effects of lockdown(s) on children prosocial behaviour and peer relationships is warranted, as the COVID-19 pandemic can represent a developmental turning point, “setting into motion accumulating advantages and disadvantages that can deflect long-term trajectories of well-being” (Benner & Mistry, 2020, p. 238).

Despite its limitations, the present study contributes to the literature on relations between HLE, out-of-home activities, prosocial behaviour and peer relationships. In continuing to gain an in-depth view on relations between HLE, out-of-home activities, and socio-emotional skills, it seems essential to consider various features of out-of-home activities as well as various facets of socio-emotional skills in future studies.

## Data Availability

The data used in this study is available for academic research under 10.5157/NEPS:SC1:8.0.1.

## Code availability

The code for the analyses will be made available upon request.
